# Supplementary material for: Two isoforms of Arabidopsis protoporphyrinogen oxidase localize in different plastidal membranes
Source: Plant Physiol. 2023 Feb 21;192(2):871–85. doi: 10.1093/plphys/kiad107 (PMC10231370; doi:10.1093/plphys/kiad107)
Supplement: kiad107_Supplementary_Data [file kiad107_supplementary_data.pdf]

Genotyping

Garlic_LB	TAGCATCTGAATTTCATAACCAATCTCGATACA
pROK_LB4	CTGGCGTAATAGCGAAGAGG
Gabi LB	CCCATTTGGACGTGAATGTAGACAC
PPO2F2	CGCATAATGAAACGCAGAGA
PPO2R2	TCGCTGAAGGTCAGAAGTCA
PPO2F3	ATTTTATGGCGATGAGCTGTTT
PPO1 down fw	TGCGCCCTCTTTCTGTAAGT
AtPPO1_genot.WtFw	TGGAGAGCGGAGGATACAAC
AtPPO1_genot.WtRv	CCGACGAAAGCAGATAGAGC
PCR I	CTCGGGGAGAAAGTTTAGC
PCR II	GTTTAGCTGAAAGGGTTGTC
PCR III	GGGTTGTCCTCCTTTCAT
PCR IV	CACATTGCACAGAGGAGC

Cloning

PPO1_5'UTR_RV_fusion	CCTGCAGGATAGGCGCGCCGGAAATCGCAGAGAATTCGG
PPO1_3'UTR_FW_fusion	GGCGCGCCTATCCTGCAGGATGTAAAACATTAAATCTCC
P1P2_Fusion_RV	CTACTGCTCCAGACGCGGCCACTGAACAACGG
P1P2_Fusion_FW	CCGTTGTTCAGTGGCCGCGTCTGGAGCAGTAG
RbcsP2_Fusion_RV	TGCTCCAGACGCGGAATCGGTAAGGTCAG
RbcsP2_Fusion_FW	CCGATTCCGCGTCTGGAGCAGTAG
PPO1_Promoter_FW_SacI	CTAGAGCTCTACAAGTGAACACGACACAA
PPO1_3'UTR_RV_PmlI	CTACACGTGAACCAAAGACCCTTCTCCTA
PPO1_FW_SacI	GAGCTCCATGGAGTTATCTCTTCTCCG
PPO1_FW_AscI	CTAGGCGCGCCATGGAGTTATCTCTTCTCCG
PPO2_FW_SacI	GAGCTCGCAATGGCGTCTGGAGCAG
PPO2_RV_SacI	GAGCTCTTATAAGCTGTCATTTGGTTTCTTG
PPO2_FW_AscI	CTAGGCGCGCCATGGCGTCTGGAGCAGTAGC
PPO2_RV_SbfI	CTACCTGCAGGTTATAAGCTGTCATTTGGTT
RbcS_FW_PmlI	ACACGTGAAAATGGCTTCCTCTATGCTC

RT-qPCR

PPO1	GCAAGATTAGGTAGCAAAGTTAAG
	GCACGGAAACTAAACCATCT
PPO2	TAATTATGACGGCTCCTCTGTG
	GGTCTCTTTACTTTCTCCTTTGTG
PPO1 (3´UTR)	AAACATTAAATCTCCCAGCTTG
	AATGAAATTGAAGTTTGATATCTCAAA
FC1	CTCCATGTCTATCTCCTCAAGTGTCT
	AGCCGTTCCAGTCAGTTTCGT
FC2	CGAGCGGAACTAACGACTGTC
	CCATACGTTGGTGCTATGGCT
PORB	TGATTACCCTTCAAAGCGTCTCA
	CAATGTATTCGTGTTCCCGGT
HEMA1	TTGCTGCCAACAAAGAAGAC
	CCGTCTCCAATGAATCCCTC
SAND	TGATTGCATATCTTTATCGCCATC
	AACTCTATGCAGCATTTGATCCACT

Supplemental Table S1

List of oligonucleotides used in the present study.
